# Professional Identity Formation – important, complex, and dynamic

**DOI:** 10.3205/zma001836

**Published:** 2026-03-23

**Authors:** Pascal O. Berberat, Susanne Michl, Jan Schildmann, Götz Fabry

**Affiliations:** 1 Technical University of Munich, TUM School of Medicine and Health, Department of Clinical Medicine TUM Medical Education Center, Munich, Germany; 2 Charité – University Hospital, Institute for the History of Medicine and Ethics in Medicine, Berlin, Germany; 3 Martin Luther University Halle-Wittenberg, Medical Faculty, Institute for History and Ethics of Medicine, Halle (Saale), Germany; 4 University of Freiburg, Faculty of Medicine, Institute for Medical Psychology and Medical Sociology, Freiburg, Germany

## Editorial

The concept of Professional Identity Formation (PIF) has increasingly become the subject of debate in German-speaking countries, particularly regarding the advancement of medical education [9], [17], [20], [23]. At the same time, even a rather shallow review of the literature gives the clear impression that the discussions differ considerably in terms of their technical perspective and the associated scientific standards. This becomes particularly evident in the question of what is meant by professional identity. Various studies from the Anglo-American context have attempted to reconstruct the development and transformation of this concept, and the aspects identified in these analyses can also be found in contributions to the German-language discourse [10], [11].

Initially, there have been several attempts to describe professional identity as an individual characteristic – as a “favorable” combination of character traits, attitudes, competencies, or virtues that enable a person to behave “professionally” in challenging situations [5], [4]. For medical education, such findings suggest, that, where possible, only those individuals who already possess the appropriate character should be admitted to medical school, and that they should then receive further training in this regard [16]. Many innovation and project reports in medical education research therefore describe interventions that are explicitly aimed at fostering these individual traits and dispositions [15]. The debate about whether non-cognitive criteria – such as empathy – should be considered in addition to cognitive criteria (e.g., GPA and medical admission tests) for admission to medical school can also be traced back to this understanding of professional identity [24].

A critical caveat to this view is its failure to take sufficient account of the influence of social and systemic factors, leaving the responsibility for professional conduct solely with individuals [14]. It is evident, however, that certain values – such as social justice in healthcare or the handling of economically driven conflicts of interest – are hardly within the exclusive control of individuals, but are at least partially shaped or even largely determined by the respective context [10]. Such an understanding also has consequences for medical education practice, which would need to place greater emphasis on contextual factors such as the hidden curriculum and encourage reflection on systemic aspects that students encounter during clerkships or clinical placements.

Furthermore, the object of discussion is defined heterogeneously and sometimes rather vaguely. Two references frequently found in the PIF literature illustrate this problem. On the one hand, there is frequent (though not always accurate) reference to Merton’s phrase “think, act, and feel like a physician” [13]. This definition, however, raises more questions than it answers. Most notably, it remains unclear what it actually means to “act like a physician.” As in many other definitional attempts, this refers to a medical ideal – or at least an abstract type – without clearly specifying the concrete attributes, norms, and values associated with it.

On the other hand, with reference to the 2010 Carnegie Foundation Report “Educating Physicians”, which is seen as the successor to the Flexner Report, PIF is defined as “the development of professional values, actions, and aspirations” as “a major focus of medical education” [3]. Even with this outcome-oriented definition, it would still be necessary to specify more precisely which professional values, actions, and aspirations are meant.

It is unlikely that this will be achieved, and in view of pluralistic, multicultural, open societies, it is hardly desirable to arrive at a definitive answer, such as a defined canon of professional standards, values, and character traits. However, this does not mean that the content of PIF is arbitrary or that it is sufficient to focus solely on acquiring competencies such as reflection (even though this is undoubtedly central to medical practice). Rather, there is a need for an intensified discussion about what constitutes good medical practice and good medical education, especially in light of the dynamic development of both healthcare and education systems [12].

Few would dispute that the concepts outlined above represent important – if not central – aspects of medical education. At the same time, it should be clear at second glance, at the latest, that PIF in such a vague concept is likely to be a gateway for very different ideas of what constitutes “good” medical training to be introduced.

Nevertheless, a concept such as PIF, even when initially heterogeneous and somewhat vague, can still make a meaningful contribution to the advancement of medical education. The discussion of values in medical education, for example, is particularly important in pluralistic societies, as is the reminder that medical education involves more than assessable knowledge and skills. In this sense, PIF places the physician as a human being at the center. Based on established frameworks, this includes the acquisition of various professional roles (communicator, team member, health advocate, manager and leader, professional, scholar, and medical expert), which together constitute medical practice through a wide range of specialized competencies [25]. Notably, this concept of competence explicitly includes a professional attitude alongside knowledge and skills [26], although this dimension is often neglected in educational practice. It can be assumed that this (mature) attitude, in combination with professional knowledge and skills, has a decisive influence on medical practice and likely also on personal and professional development, sense of meaning, and mental health in the medical profession.

At the same time, however, it is also clear that more is needed in terms of conceptually refined discussions and empirical studies. In both cases, clarification and specification are necessary [25]. In addition to discussing content, further clarification is needed regarding how the process or development of PIF itself can be better understood. This includes a closer examination of the terminology used in this context, including the question of how the English term “identity formation” should best be translated.

The contributions in this issue can be grouped into four thematic areas corresponding to the dimensions outlined above. The first four articles address theoretical foundations and aspects of conceptual definition. Schumm et al. [22] provide a literature review analyzing international definitions of PIF and identifying central elements such as values, norms, and the goal of “thinking, acting, and feeling like a physician.” Schick et al. [19] present a consensus-based conceptualization for the German-speaking context, describing PIF as an ongoing (conscious and unconscious) interaction process between person and environment. Ahles et al. [1] examine the integration of PIF into the National Competency-Based Learning Objectives Catalogue for Medicine (NKLM 2.0) and identify essential learning objectives, particularly related to role understanding and self-reflection. Wild [30] expands the normative foundation of PIF by developing an “ethos of connectedness” and explicitly extending medical responsibility to include ecological and societal dimensions.

The following three contributions present curricular implementations and longitudinal approaches. Gehlhar etal. [8] show that acceptance of a longitudinal “Professional Development” pathway in Oldenburg increased following curricular reforms and that its success depends particularly on the quality of tutorial support. Drossard et al. [6] demonstrate, using the “Maturitas” curriculum at the University of Augsburg, that mentoring and specific learning interventions systematically promote the transition into the medical role; while reflective abilities increase, fluctuating participation rates remain a challenge. Schumm et al. [21] integrate the CanMEDS roles into a clinical lecture series and use interactive methods such as panel discussions and photography to stimulate early identity development.

Four additional articles focus on specific teaching formats and topic areas. Schick et al. [18] prepare students in their final year with the seminar “Suddenly in the Clinic,” using case-based learning and storytelling to address complex clinical dilemmas. Vogel et al. [27] sharpen understanding of other health professions through simulations of ethical case consultations in interprofessional groups. Wald et al. [28] use the confrontation with medical practice in Nazi Germany as a catalyst for moral development and value-based identity, which in the long term strengthens the understanding of one’s roles as a responsible member of society. Warnken et al. [29] establish a student peer-support system that integrates self-care and collegial support into professional identity and addresses stressors such as exam anxiety and performance pressure during medical school to strengthen the resilience of future physicians.

Finally, two studies address the assessment of individual factors in the context of PIF. Fey et al. [7] present the “MediProf” questionnaire, a reliable German-language self-assessment instrument measuring professionalism across four dimensions: toward oneself, patients, colleagues, and society. Albrecht et al. [2] identify types of students – from the “called” to the “doubters” – and show how experiences of meaning and crises of meaning are closely linked to study motivation.

This special issue, initiated by the GMA Committee on Professional Identity Formation, does not resolve the challenges outlined at the outset. However, it clearly resonates with ongoing discussions and activities at medical schools in German-speaking countries. As a result, this special issue includes conceptual, empirical, and practice-oriented work that, at least in our opinion, provides a good overview of the current state of research and teaching on this topic. As editors, we were somewhat overwhelmed – in multiple respects – by the number and diversity of submissions. We thank all authors for their manuscripts and the many reviewers for their careful evaluations, which were not always easy given differing disciplinary perspectives. Finally, we hope that readers will find these contributions valuable and that they may, in the future – and ideally also within our committee – contribute to the further development of PIF in undergraduate, postgraduate, and continuing education in the health professions.

## Authors’ ORCIDs

- Pascal O. Berberat: [0000-0001-5022-5265]
- Susanne Michl: [0000-0001-8387-2434]
- Jan Schildmann: [0000-0002-5755-7630]
- Götz Fabry: [0000-0002-5393-606X]

## Competing interests

The authors declare that they have no competing interests. 
